# Sustainable Au (III) adsorption using greenly Fe-BTC/PpPDA synthesized composite: kinetic and isothermal studies

**DOI:** 10.1098/rsos.251604

**Published:** 2026-09-09

**Authors:** Woulou Somda, Francis O. Konate, Konan R. Koffi, Gerard Y. Sanou, Rafiatou Lingane, Isaï N. Bombiri, Mohamed Khalid, Arsène H. Yonli

**Affiliations:** ^1^ Université Joseph Ki-Zerbo, Ouagadougou, Burkina Faso; ^2^ Université Félix Houphouët-Boigny, Abidjan, Abidjan Autonomous District, Côte d'Ivoire; ^3^ LPO Edouard Vaillant, France

**Keywords:** green synthesis, characterization, Fe-BTC/PpPDA, gold adsorption

## Abstract

A novel porous composite material, Fe-BTC/PpPDA, was synthesized via a green solvothermal route by integrating polyparaphenylenediamine (PpPDA) into the Fe-BTC metal–organic framework. The material was characterized by X-ray diffraction, FTIR, ultraviolet-visible and scanning electron microscopy/energy-dispersive X-ray spectroscopy analyses, confirming successful incorporation of nitrogen-rich PpPDA into the metal organic frameworks structure. The composite exhibited a well-developed porous morphology and abundant functional groups favourable for metal binding. Batch adsorption experiments demonstrated a high gold uptake capacity (*q*_max_ = 1045.8 mg g^–1^) with rapid kinetics, reaching equilibrium within 30 minutes. The adsorption behaviour followed the Langmuir model (R^2^ = 0.9847), indicating monolayer adsorption and high selectivity. These findings position Fe-BTC/PpPDA as an efficient, sustainable and selective adsorbent for gold recovery from polymetallic aqueous solutions.

## 1. Introduction

Metal organic frameworks (MOFs) are a class of porous hybrid materials renowned for their large surface areas, tunable structures and versatile functionalities, making them attractive for applications such as catalysis, gas storage, separation and adsorption [1–5]. Among MOFs, iron-based materials like Fe-BTC (iron(III) trimesate) are of particular interest owing to their low cost, environmental compatibility and scalability, especially in water purification and resource recovery [6–8].

The selective recovery of gold from industrial effluents and electronic waste has become increasingly important owing to economic and environmental concerns. However, conventional gold extraction methods often rely on toxic reagents or energy-intensive processes, limiting their sustainability. In this context, MOF-based adsorbents provide a promising alternative, especially when modified to improve selectivity and adsorption capacity [9,10].

One effective strategy involves hybridizing MOFs with functional polymers. Conductive polymers such as polyparaphenylenediamine (PpPDA), rich in nitrogen-containing groups, exhibit strong coordination with metal ions via chelation and π-π interactions [11–13]. Incorporating such polymers into MOFs can enhance their adsorption performance by introducing additional active sites and modulating porosity [14,15].

In this study, we report the green solvothermal synthesis of an Fe-BTC/PpPDA composite and investigate its structural, morphological and optical properties. The optical properties of ultraviolet-visible (UV-Vis) are studied to deduce electronic changes during polymerization or coordination of gold ions [16]. Batch adsorption studies reveal the composite's exceptional affinity for ${AuCl}_{4}^{-}$ species, characterized by a remarkable maximum adsorption capacity of 1045.8 mg g^–1^ and a notably fast equilibrium time of 30 minutes. The strong correlation with the Langmuir isotherm model (R^2^ = 0.9847) suggests a favourable, monolayer-type adsorption mechanism conducive to selective metal recovery. Motivated by this promising performance in gold recovery from complex aqueous matrices, we now turn to the experimental framework underpinning these findings. The ensuing Material and methods section delineates the synthesis route, composite characterization, and the specific parameters of the batch adsorption experiments that substantiate this high efficiency.

## 2. Material and methods

### 2.1. Material

Iron(III) chloride hexahydrate (FeCl_3_.6H_2_O, 97%) was purchased from AWR Chemicals. Trimethyl-1,3,5-benzenetricarboxylate (C_6_H_3_(CO_2_CH_3_)_3_, 98%), paraphenylenediamine (C_6_H_4_(NH_2_)_2_, 99%) and methanol (CH_3_OH, 99.9%) were obtained from Sigma-Aldrich. All reagents were of analytical grade and used as received. Synthesis was carried out using a Teflon-lined autoclave reactor. In adsorption experiments, an aqueous gold (III) chloride solution is employed.

### 2.2. Methods

#### 2.2.1. Hydrothermal synthesis of Fe-BTC

Fe-BTC was synthesized using a hydrothermal method in a Teflon-lined autoclave. Iron (III) chloride hexahydrate was reacted with trimethyl-1,3,5-benzenetricarboxylate in an aqueous solvent to yield the Fe-BTC MOF, as illustrated in equation (2.1). In a typical procedure, 5.0 g of trimethyl-1,3,5-benzenetricarboxylate and 14.46 g of FeCl_3_.6H_2_O were dissolved in 200 ml of distilled water [14,17]. The mixture was transferred to the reactor and heated at 150°C for 72 hours. After cooling to room temperature, the solid product was filtered under vacuum and washed thoroughly with distilled water and methanol. The obtained solid was air-dried for 24 hours, followed by purification using Soxhlet extraction with methanol for an additional 24 hours. This step is crucial for product purity as it removes excess soluble reactants. The material was then dried at room temperature for 12 hours and subjected to a final thermal treatment at 150°C for 24 hours. The final product, an orange powder, was identified as Fe-BTC.
(2.1)
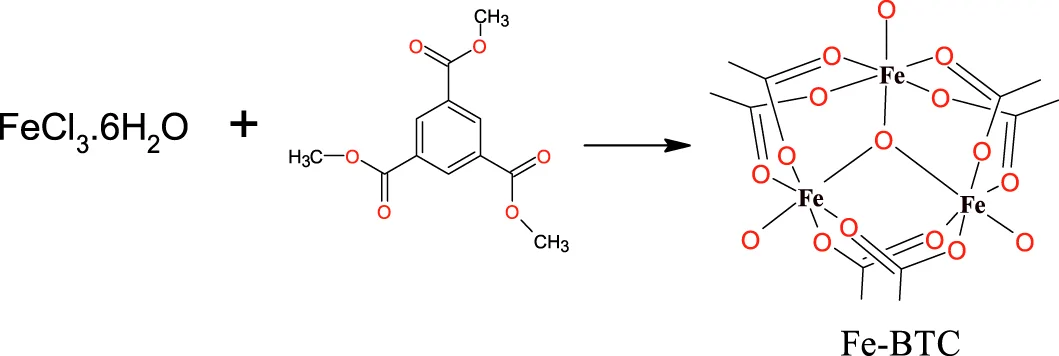


### 2.2.2. Solvothermal synthesis of Fe-BTC/PpPDA

The composite Fe-BTC/PpPDA was synthesized in diluted methanol. Thermal activation of Fe-BTC allowed it to react with pPDA, which underwent *in situ* polymerization, leading to the formation of the composite, as shown in equation (2.2). Specifically, 3 g of thermally activated Fe-BTC was dispersed in 500 ml of methanol (0.02 mol.l^–1^) and 0.802 g of pPDA was added [14,18]. The mixture was stirred magnetically at room temperature for 24 hours. The resulting solid was filtered under vacuum and washed thoroughly with distilled water and methanol. After air drying for 24 hours, the solid was purified by Soxhlet extraction using methanol for 24 hours. Finally, the purified product was dried for 12 hours at room temperature and subjected to thermal treatment at 150°C for 24 hours [19]. The final product, a purple powder, was identified as Fe-BTC/PpPDA:

(2.2) Fe−BTC + n(pPDA) → Fe−BTC/PpPDA.

### 2.2.3. Characterization of Fe-BTC and Fe-BTC/PpPDA

The structural, morphological and compositional properties of Fe-BTC and Fe-BTC/PpPDA were characterized using several analytical techniques [20]: (i) X-ray diffraction (XRD) was performed using a SIEMENS D5000 THETA diffractometer equipped with a copper anode (λ = 1.5406 Ǻ) to determine crystal structure and phase purity. (ii) Infrared (IR) spectroscopy was carried out with a Shimadzu FTIR-8400S spectrometer in the range 400–4000 cm^–1^ to identify functional groups. (iii) UV-Vis spectroscopy was conducted between 190 and 900 nm using a Shimadzu UV-2101PC spectrometer, with tetrahydrofuran (THF) as solvent [21]. (iv) Scanning electron microscopy (SEM) coupled with energy-dispersive X-ray spectroscopy (EDS) was used to analyse morphology, surface structure and elemental composition; these measurements were performed using a HITACHI SU8020 microscope.

### 2.2.4. Adsorption experiments

Batch adsorption experiments were carried out to study the kinetics of ${AuCl}_{4}^{-}$ ion adsorption onto Fe-BTC/PpPDA composite [22]. 20 mg of Fe-BTC/PpPDA was dispersed in 50 ml of an aqueous solution containing ${AuCl}_{4}^{-}$ ions with an initial concentration (*C₀* = 10 mg l^–1^) at room temperature. The suspension was stirred at constant speed, and aliquots were withdrawn at specific time intervals (0, 5, 10, 15, 20, 25, 30, 60, 90, 120 and 180 minutes). The remaining ${AuCl}_{4}^{-}$ concentration was measured by atomic absorption spectrometry. The amount of gold adsorbed at time *t* (*q_t_*) was calculated using [23]:

(2.3) $$q_{t}\;=\left(c_{0}-c_{t}\right)\frac{V}{m}.$$

Isotherm studies were conducted by varying the initial ${AuCl}_{4}^{-}$ concentration under optimized pH = 2 [24] and contact time conditions (30 minutes). After equilibrium, the residual concentration (*C_e_*) was determined, and the equilibrium adsorption capacity (*q_e_*) was calculated. The data were modelled using Langmuir and Freundlich isotherms [25,26]:

Langmuir equation:

(2.4) $$\theta =\;\frac{q_{e}}{q_{m}}=\;\frac{K_{L}\;\times \;C_{e}}{1+K_{L}\;\times \;C_{e}}$$

and Freundlich equation:

(2.5) $$q_{e}=\;K_{F}\;\times \;C_{e}^{\frac{1}{n}}.$$

The Langmuir model was used to determine *q*_max_ and adsorption affinity *K_L_*.

## 3. Results and discussion

### 3.1. Synthesis

**Figure 1.**
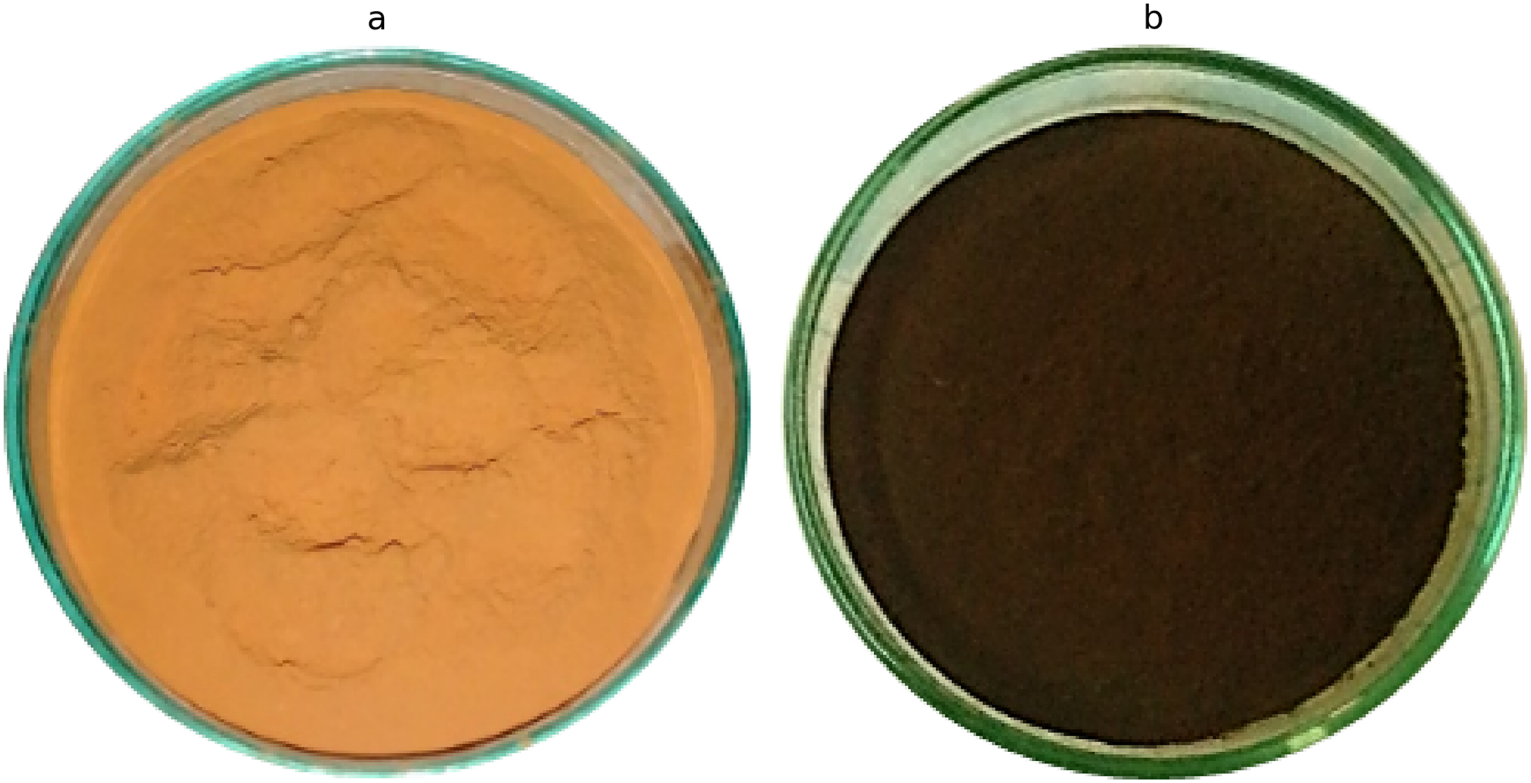
(a) Orange powder of Fe-BTC. (b) Purple powder of Fe-BTC/PpPDA.

The synthesized Fe-BTC and Fe-BTC/PpPDA materials were obtained as fine powders (figure 1), each exhibiting melting points above 300°C. This contrasts with previous reports describing amorphous structures with melting points below 200°C [27–29]. This remarkable thermal stability, especially when compared with certain MOFs that degrade below 150°C, can be attributed to both the synthesis conditions and the choice of organic ligands [30]. Fe-BTC was synthesized under a pressure exceeding 7.5 bars. Although the same synthesis can be performed at atmospheric pressure, it would require more than 72 hours to complete. High-pressure synthesis not only accelerates the reaction but also promotes the formation of rigid frameworks with enhanced thermal resistance. The combined use of hydrothermal and solvothermal techniques proved effective in producing robust MOF structures. Additionally, these methods are considered environmentally friendly, as they generate minimal waste and use relatively benign solvents [8].

### 3.2. Characterization

#### 3.2.1. X-ray diffraction

**Figure 2.**
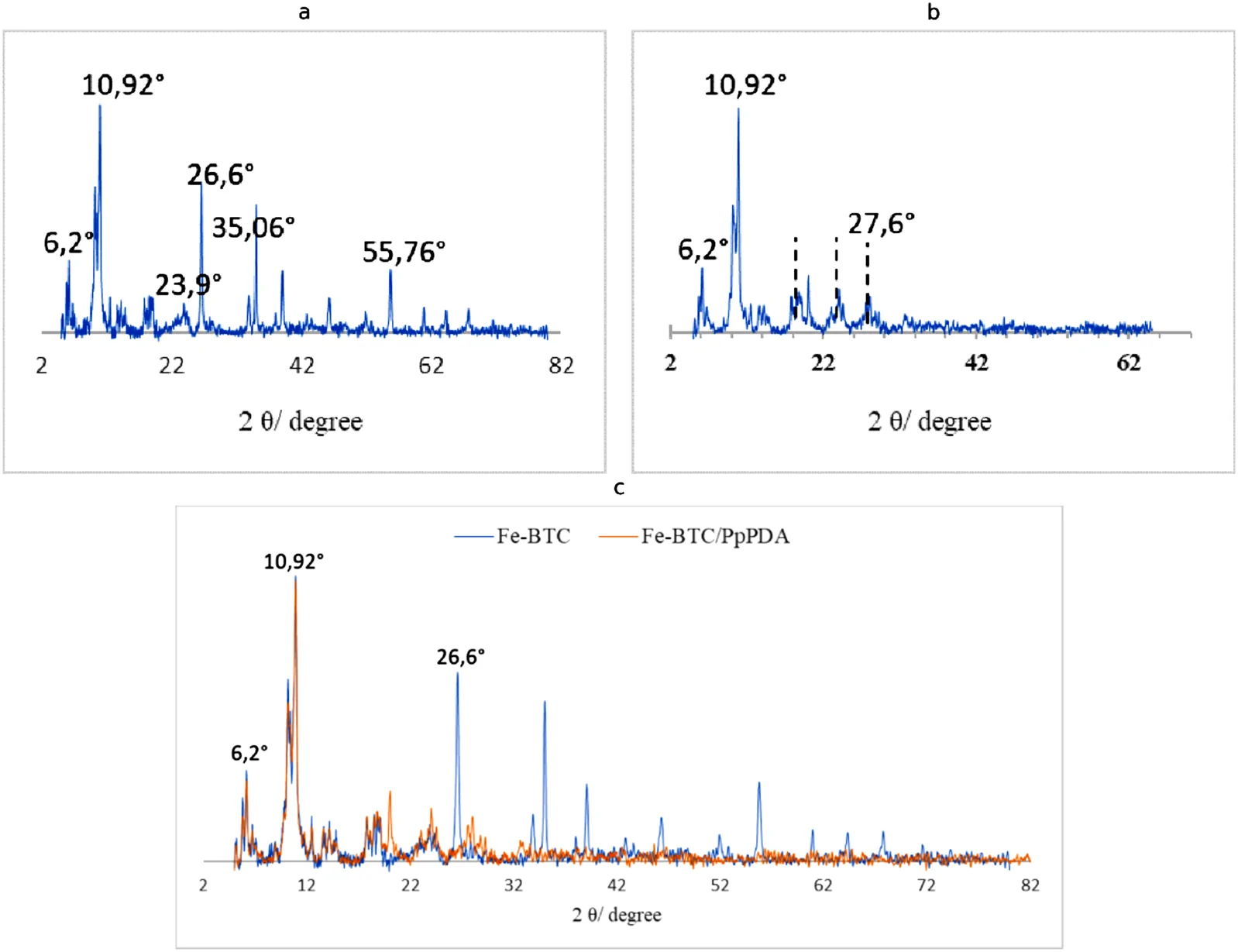
(a) XRD pattern of Fe-BTC. (b) XRD pattern of Fe-BTC/PpPDA. (c) Superimposed XRD patterns of Fe-BTC and Fe-BTC/PpPDA.

The X-ray diffraction (XRD) patterns of the synthesized MOFs Fe-BTC and Fe-BTC/PpPDA (figure 2a,b) display distinct diffraction peaks indicative of crystalline phases at specific 2θ angles. The Fe-BTC sample exhibited well-defined peaks at 6.2°, 10.92° and 23.9°, corresponding to characteristic crystallographic planes of its structure as reported in the literature [14,31]. The sharpness and clarity of these peaks confirm the material's crystalline nature and enable the identification of its phase composition. Additional peaks at 26.6°, 35.06° and 55.76° are attributed to Fe-O bonding structures [31]. By contrast, the XRD pattern of Fe-BTC/PpPDA showed broader and less intense peaks, indicating a lower degree of crystallinity in the composite material. The composite exhibited diffraction peaks at 2θ values of 6.2°, 10.92° and 27.6° [32]. Following the incorporation of pPDA into the Fe-BTC framework, a significant reduction in the intensity of the peaks at 27°, 35.06° and 55.76° was observed [14,33], while the intensity of the 10.92° peak increased (figure 2c). These changes in peak intensity may result from internal structural reorganization of the Fe-BTC matrix owing to interaction with pPDA and/or the formation of the poly(p-phenylenediamine) polymer [34]. A slight shift in the peak at 26.6° was also noted. The persistence of Fe-BTC's characteristic peaks in the Fe-BTC/PpPDA pattern confirms that the Fe-BTC structure remains intact in the composite. Moreover, additional peaks at 20.14° and 24.14°, which appear after pPDA incorporation, are associated with the polymer structure [35].

#### 3.2.2. Infrared spectroscopy

**Figure 3.**
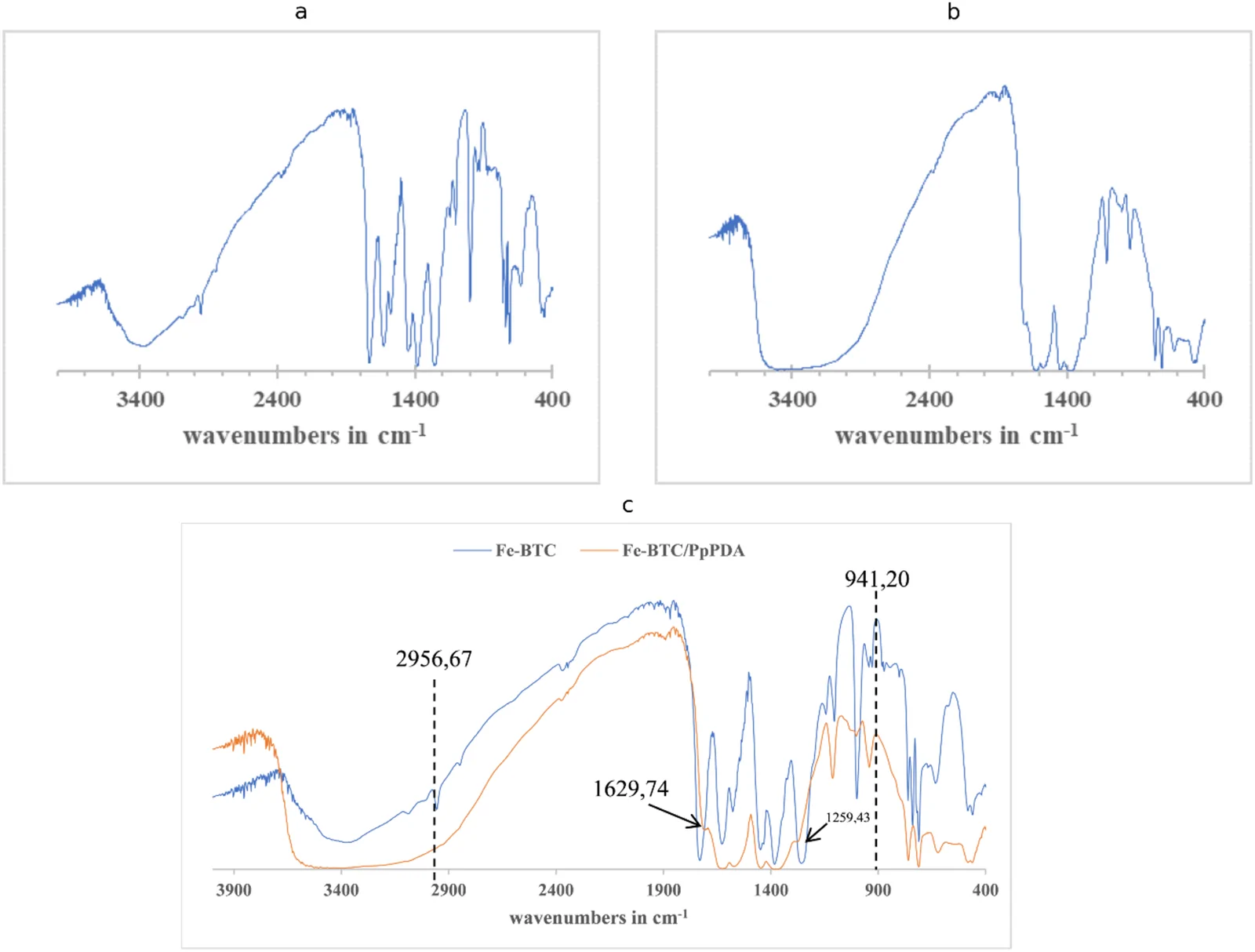
(a) Infrared spectrum of Fe-BTC. (b) Infrared spectrum of Fe-BTC/PpPDA. (c) Superimposed spectra of Fe-BTC and Fe-BTC/PpPDA.

The IR spectra of Fe-BTC and Fe-BTC/PpPDA (figure 3a,b) revealed various vibrational bands corresponding to functional groups from the precursor molecules, as well as new bonds formed during synthesis. In the region between 400 and 1000 cm^–1^, deformation vibrations of C-H and C-C bonds both in-plane and out-of-plane are observed in both materials. Between 1000 and 1600 cm^–1^, stretching vibrations corresponding to C-C, C-O, C-N and C=N bonds are detected. The replacement of trimesic acid with an aromatic triester in Fe-BTC synthesis did not significantly alter the IR spectrum. In the Fe-BTC/PpPDA spectrum, characteristic stretching vibrations of single C-C and C-N bonds appear near 1200  and 1300 cm^–1^, respectively. Vibrations from double bonds (C=C and C=N) are observed above 1400 cm^–1^. Both materials show a broad absorption band in the 3000–3700 cm^–1^ range, corresponding to O-H and N-H stretching vibrations [32,36]. The coordination between Fe (III) centres and carboxylate groups is evidenced by absorption bands at 550 cm^–1^ and 603 cm^–1^ [37]. A band at 1604 cm^–1^ is assigned to the asymmetric stretching of carboxylate groups. Additional vibrational bands related to the aromatic ring include C-C and C-H stretching at 1396 and 710 cm^–1^, respectively. C-O and C=O stretching vibrations are observed at 1381 and 1627 cm^–1^ [2,12].

Hydrogen bonding interactions between the polymer (poly(p-phenylenediamine)) and Fe-BTC are evidenced by spectral changes, including a decrease in intensity at 1259.43 and 1629.74 cm^–1^, an increase at 941.20 cm^–1^ and the disappearance of the band at 2956.67 cm^–1^(figure 3c). These changes are attributed to hydrogen bonds between the polymer's N-H groups and the Fe-BTC oxygen atoms, as well as Van der Waals interactions between the two components. Poly(p-phenylenediamine) is characterized by distinct absorption bands at 3422, 3156, 1569, 1035 and 803 cm^–1^ [14]. Table 1 summarizes the major IR absorption bands observed in both Fe-BTC and Fe-BTC/PpPDA and their corresponding assignments.

**Table 1.** Wavenumbers of the notable vibration bands of Fe-BTC and Fe-BTC/PpPDA.

Fe-BTC/PpPDA (wavenumbers in cm^–1^)	Fe-BTC (wavenumbers in cm^–1^)	allotment
462,88 m	462,88 f	***ß*_C-C_**
621,17 f	632,61 f	***γ*_C-C_**
711,68 m	711,68 m	***ß*_C-H_**
	738,69 m	
759,90 m	761,83 m	
941,20 m	941,20 f	***ß*_N-H_**
	1000,99 m	
1110 m	1110 f	***ν*_C-C_**
	1137 f	
1259,43 Tf	1259,43 F	***ν*_C-C_**
1380,94 f	1382,87 m	***ν*_C-O_**, ***ν*_C-N_**
1446,51 f	1448,44 m	***ν*_C=C_, *ν*_C=N,_ *ß*_N-H_, *ß*_C-H_**
1571,88 f	1577,66 m	
1629,74 f	1627,81 m	
1730,03 Tf	1730,03 F	***ν*_C=O_**
	2896 f	
	2956,67 f	***ν*_C-H_**
	3080 f	
3000–3500 m	3000–3800 m	***ν*_O-H_ et *ν*_N-H_**

*υ* = elongation vibration, f = low, Tf = very low.

*ß* = deformation vibration in the plane, F = strong, m = average.

*γ* = out-of-plane deformation vibration.

#### 3.2.3. UV-Vis spectroscopy

**Figure 4.**
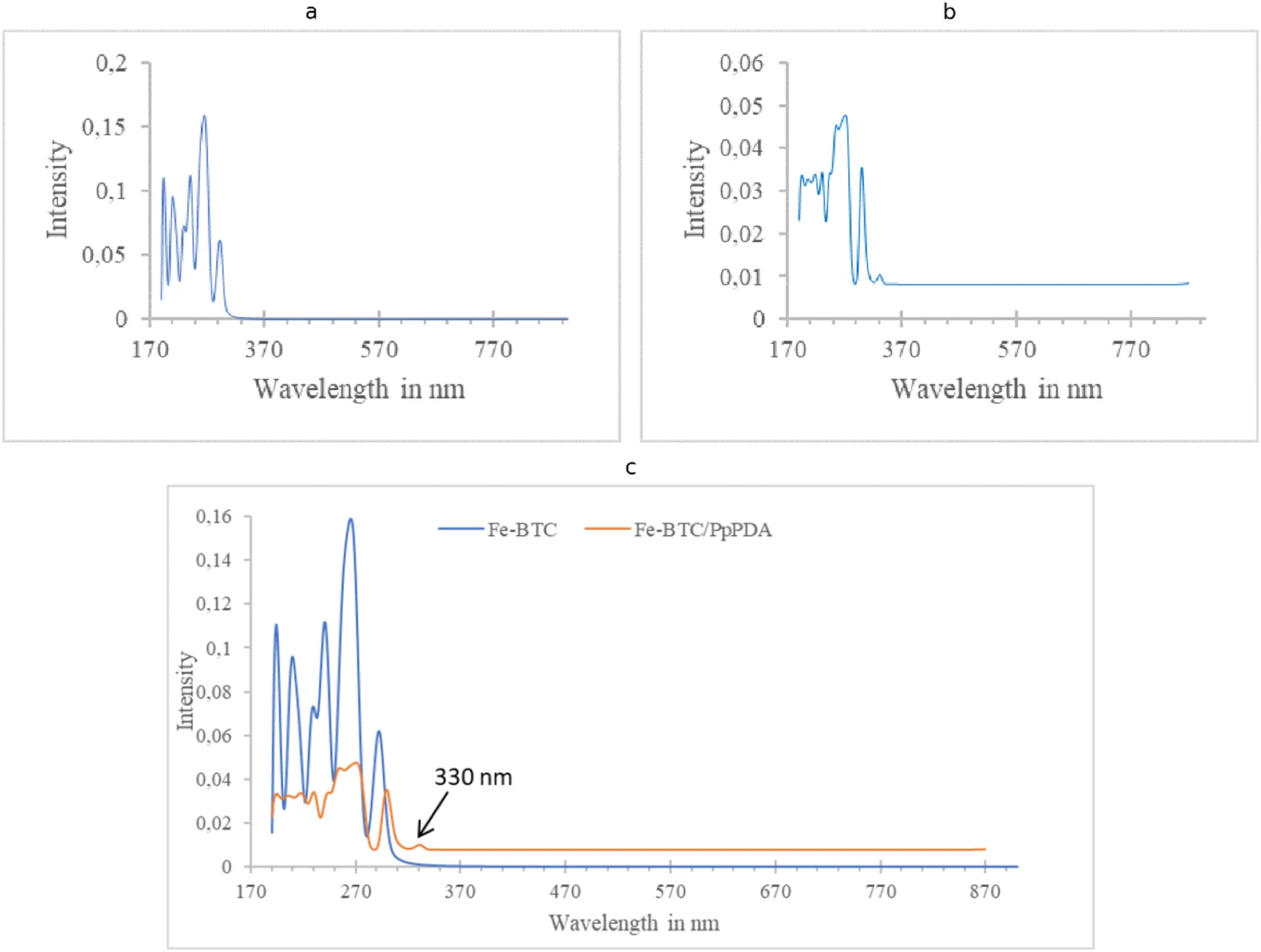
(a) UV-Vis spectrum of Fe-BTC in THF. (b) UV-visible spectrum of Fe-BTC/PpPDA in THF. (c) Superimposed spectra of Fe-BTC and Fe-BTC/PpPDA.

The UV-Vis spectra of Fe-BTC and Fe-BTC/PpPDA, recorded in THF, display absorption bands primarily located in the ultraviolet region (190–370 nm) (figure 4a,b). No significant absorption was observed beyond 370 nm, indicating that both materials are not responsive to visible light wavelengths. In the spectrum of Fe-BTC, a prominent absorption band appears at 272 nm, which is attributed to π-π* transitions of the organic ligands (BTC) and ligand-to-metal charge transfer involving Fe (III) centres. Additional absorption features include bands at 254 nm (aromatic ring), 204 nm (carboxyl groups) and 208 nm (amine functionalities). The introduction of paraphenylenediamine led to its *in-situ* polymerization, catalysed by Fe-BTC and forming the Fe-BTC/PpPDA composite. This modification affected the UV-Vis profile, resulting in a decrease in the overall absorption intensity and a shift of the absorption band at 290 nm towards longer wavelengths (bathochromic shift), as shown in figure 4. A new low-intensity absorption band appeared at 330 nm in the Fe-BTC/PpPDA spectrum. This band is characteristic of poly(paraphenylenediamine) and confirms the successful incorporation and polymerization of pPDA within the Fe-BTC framework [33].

#### 3.2.4. Scanning electron microscopy

**Figure 5.**
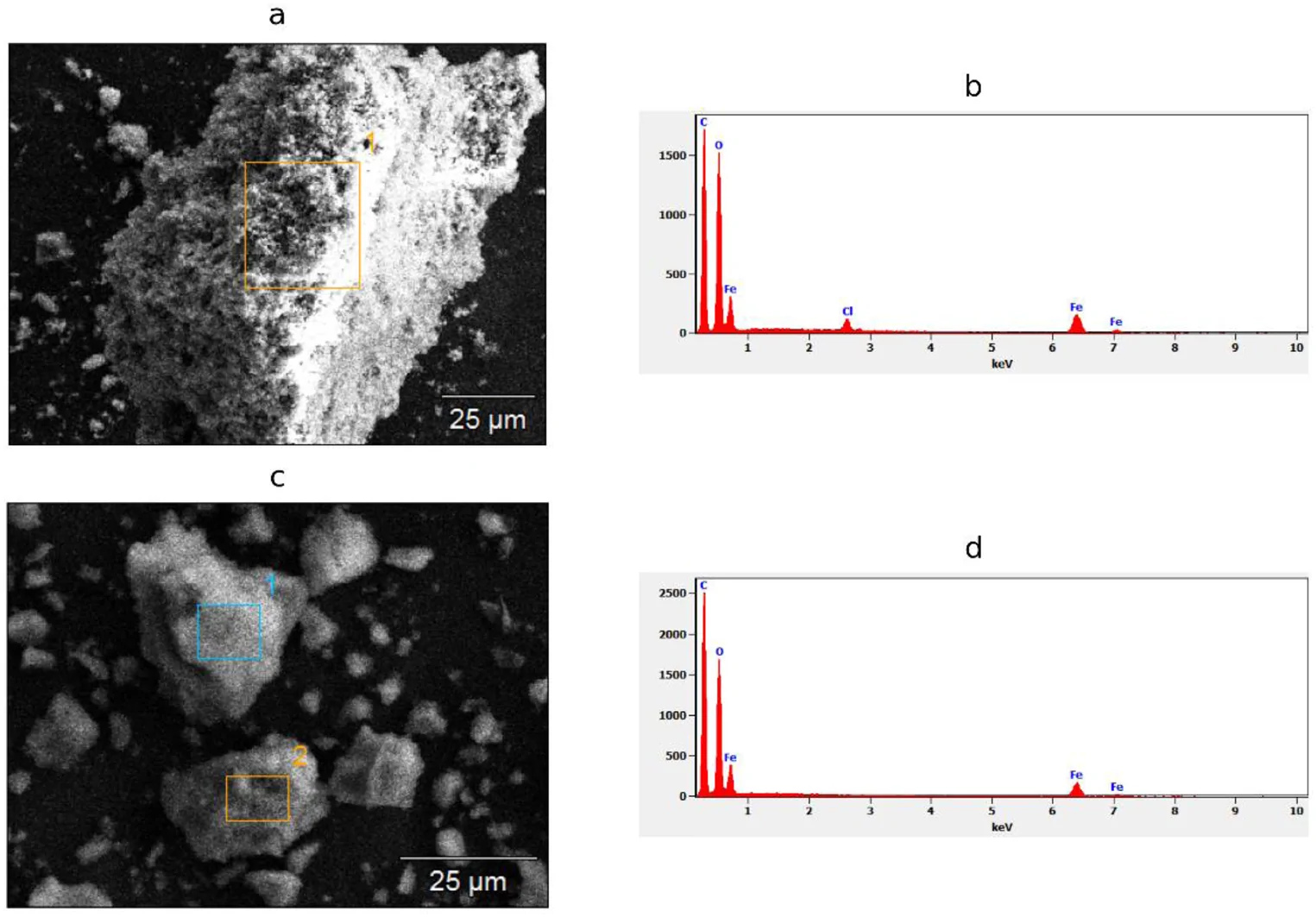
(a) SEM image of Fe-BTC. (b) EDS spectrum of Fe-BTC. (c) SEM image of Fe-BTC/PpPDA. (d) EDS spectrum of Fe-BTC/PpPDA.

Figure 5 presents the SEM images of the Fe-BTC and Fe-BTC/PpPDA materials. The SEM micrograph of Fe-BTC (figure 5a) reveals aggregates of coalesced grains, lacking well-defined crystal morphology. This appearance is indicative of the material's relatively low crystallinity, which is consistent with the XRD observations. The corresponding EDS analysis (figure 5b) confirms the presence of the expected elements carbon (C), oxygen (O), iron (Fe) and chlorine (Cl) originating from the starting reagents used in the synthesis of Fe-BTC. Similarly, the SEM image of Fe-BTC/PpPDA (figure 5c) shows agglomerates of fine grains without distinct or ordered crystalline structure. The EDS spectrum of this composite (figure 5d) reveals the presence of carbon, oxygen and iron, while chlorine is no longer detected. This absence of chlorine is attributed to the polymerization of para-phenylenediamine, which likely displaced or replaced chloride anions during composite formation. Both Fe-BTC and Fe-BTC/PpPDA exhibit morphologies characterized by irregular particle aggregation and poorly crystalline appearance, which aligns with structural data obtained from XRD analysis [14].

### 3.3. Adsorption of gold

#### 3.3.1. Effect of contact time

**Figure 6.**
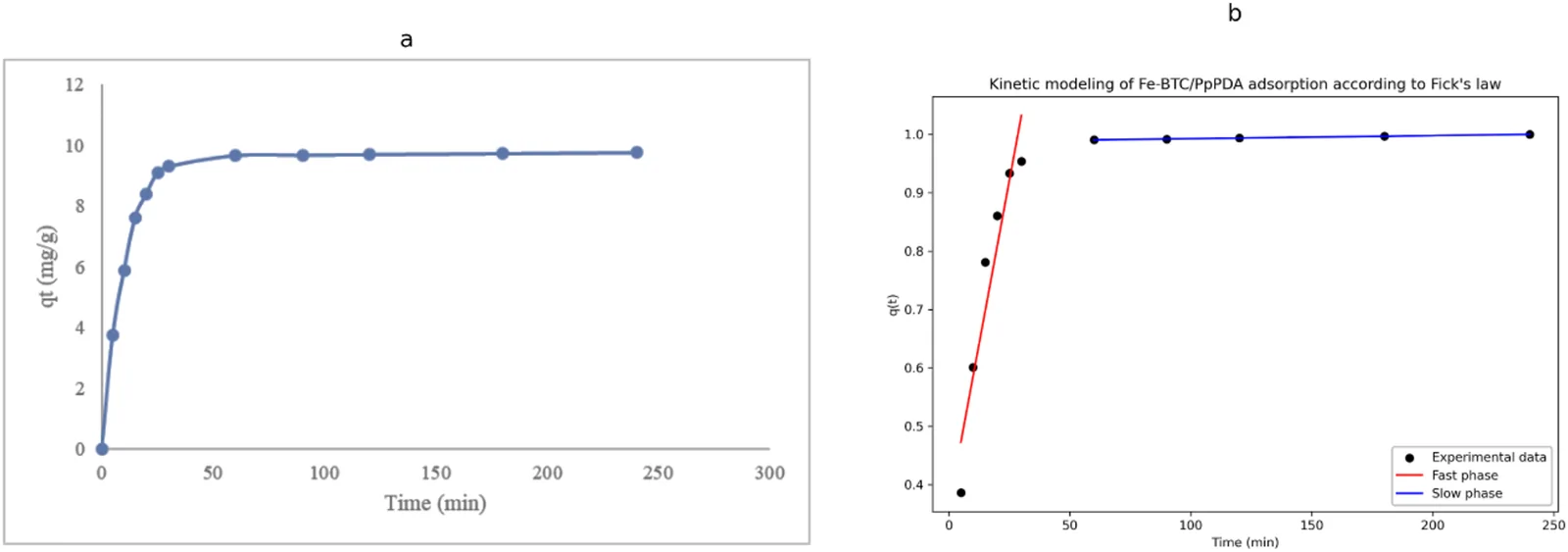
(a) Kinetic adsorption curve of Au^3+^ onto Fe-BTC/PpPDA. (b) Kinetic modelling of Fe-BTC/PpPDA adsorption according to Fick's law.

Figure 6a illustrates the adsorption kinetics of gold onto the Fe-BTC/PpPDA MOF over a period of 0 to 300 minutes. The kinetic fitting based on Fick's law reveals two distinct phases (figure 6b). The first is a rapid phase, during which gold adsorption occurs quickly within the first 10 minutes and reaches equilibrium around 30 minutes. At the beginning of the process, all active sites are available for gold atom adsorption, which accelerates the uptake. As the adsorption progresses, the concentration of gold in the reaction medium gradually decreases. Consequently, the kinetic curve reaches equilibrium, beyond which a plateau is established which corresponds to the second, slower phase. As the number of available active sites decreases significantly, the rate of gold adsorption also drops, resulting in the plateau. Fick's law describes the flux of gold ions owing to a gradient in their concentration and made it possible to determine the diffusion coefficient which is 3.05 × 10^–15^ m^2^ s^–1^. The value of this diffusion coefficient is very low, which shows surface diffusion and very narrow porosities [38,39]. The modelling of the adsorption kinetics of Fe-BTC/PpPDA gives a chemical kinetic order of 2. This pseudo-second-order kinetic model highlights a surface phenomenon. This aligns with the diffusion coefficient value, indicating low diffusion of gold ions within the pores. The 30-minute adsorption period is considered optimal for the experiments performed. This equilibrium time is higher compared with other studies that report relatively shorter durations [14]. This discrepancy may be attributed to the use of trimethylbenzenetricarboxylate as the ligand in Fe-BTC synthesis and to differences in experimental conditions.

#### 3.3.2. Adsorption isotherm

**Figure 7.**
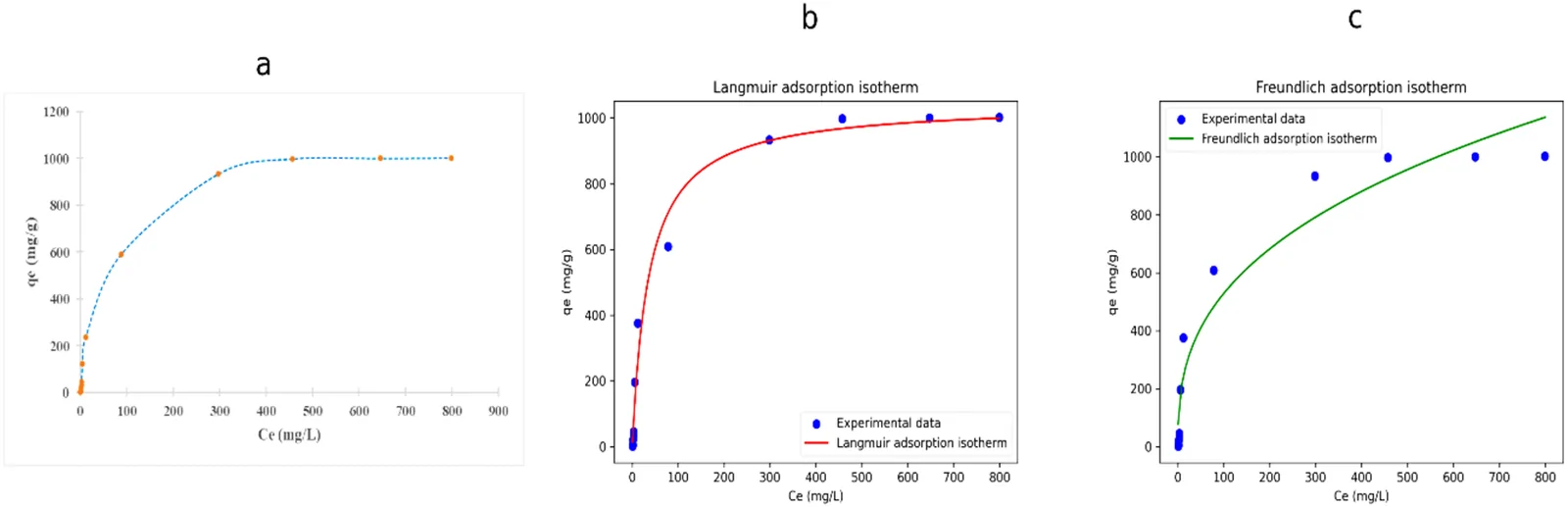
(a) Adsorption isotherm of Fe-BTC/PpPDA. (b) Langmuir isotherm. (c) Freundlich isotherm.

The adsorption isotherm of Fe-BTC/PpPDA (figure 7a) was modelled using the Langmuir and Freundlich (figure 7b,c) equations to determine the maximum gold adsorption capacity. This capacity is influenced by parameters such as solution pH and initial gold concentration.

**Figure 8.**
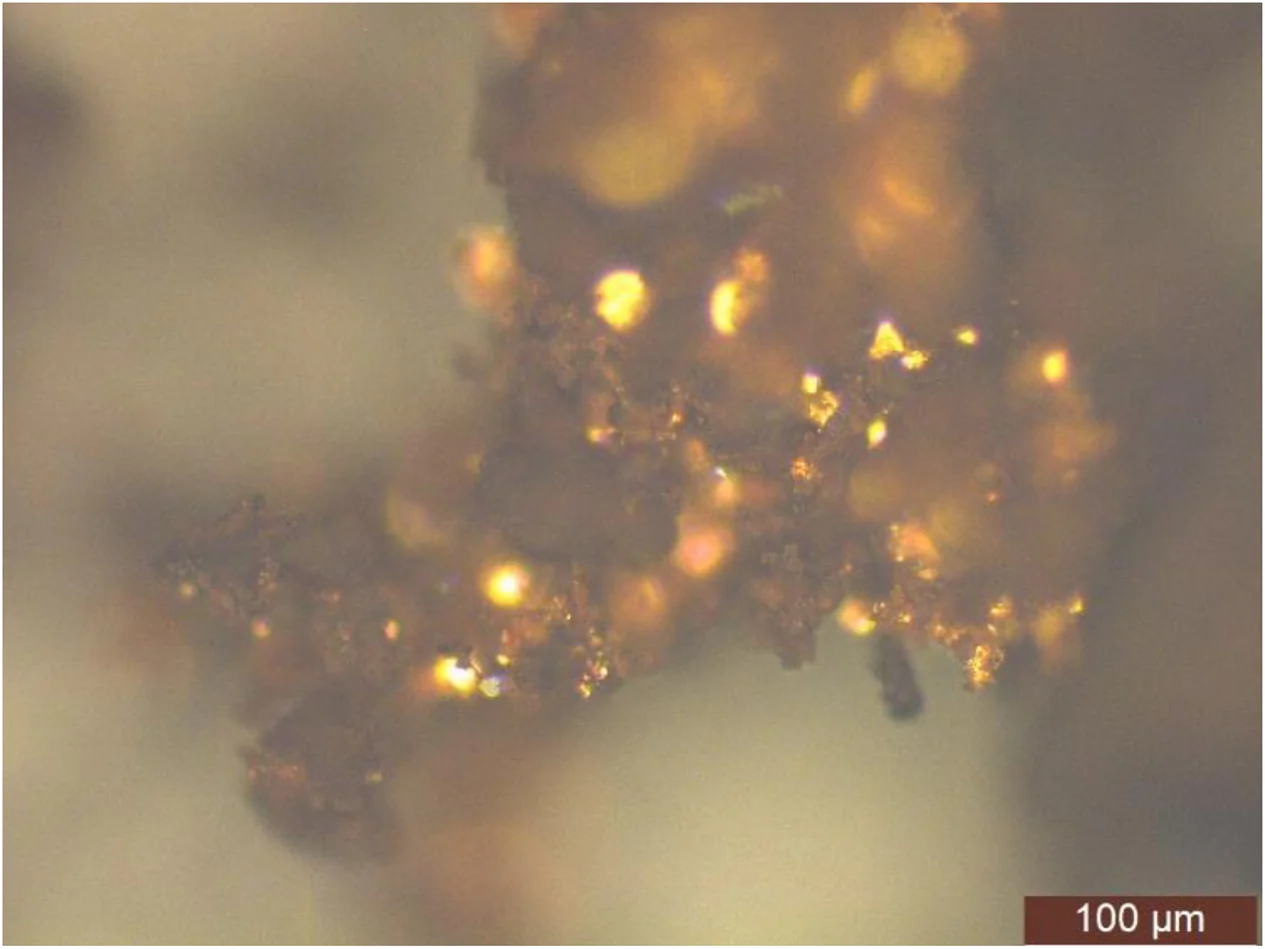
Gold recovery by Fe-BTC/PpPDA.

The fitting results (table 2) show that adsorption is favourable, as indicated by a Freundlich constant *n* > 1, and the Langmuir separation factor (*R_L_*) between 0 and 1. The low adsorption constant suggests a physisorption mechanism and the low affinity between the composite MOF and the adsorbed gold ions. Among the two models, only the Langmuir isotherm yields a maximum adsorption capacity (*q*_max_) of 1045.8 mg g^–1^, which significantly exceeds that of conventional adsorbents like activated carbon. The average fitting errors are of the same order of magnitude as the adsorbed quantities, supporting the reliability of the models. At low concentrations, high-energy sites are preferentially occupied, leading to a Langmuir-type behaviour characterized by a linear initial region followed by rapid saturation. At higher concentrations, site heterogeneity becomes evident, and lower-energy sites are also occupied, resulting in a deviation from Langmuir behaviour and better alignment with the Freundlich model. Post-adsorption analysis of the Fe-BTC/PpPDA composite MOF under optical microscopy revealed that the adsorbed gold is present in its metallic form (figure 8). Furthermore, the formation of metallic gold crystals suggests that redox reactions occur during the adsorption process. Gold ions are reduced, while the amine groups of polyparaphenylenediamine are oxidized to imine groups.

**Table 2.** Fitting parameters of adsorption models.

	fitting results					
	K (l mg^–1^)	N	Qm (mg g^–1^)	R_L_	R^2^	RMSE
Langmuir	0.0272		1045.829	0.02–0.94	0.9847	52.2333
Freundlich		2.716	97.030		0.9398	103.7866

#### 3.3.3. pH effect

**Figure 9.**
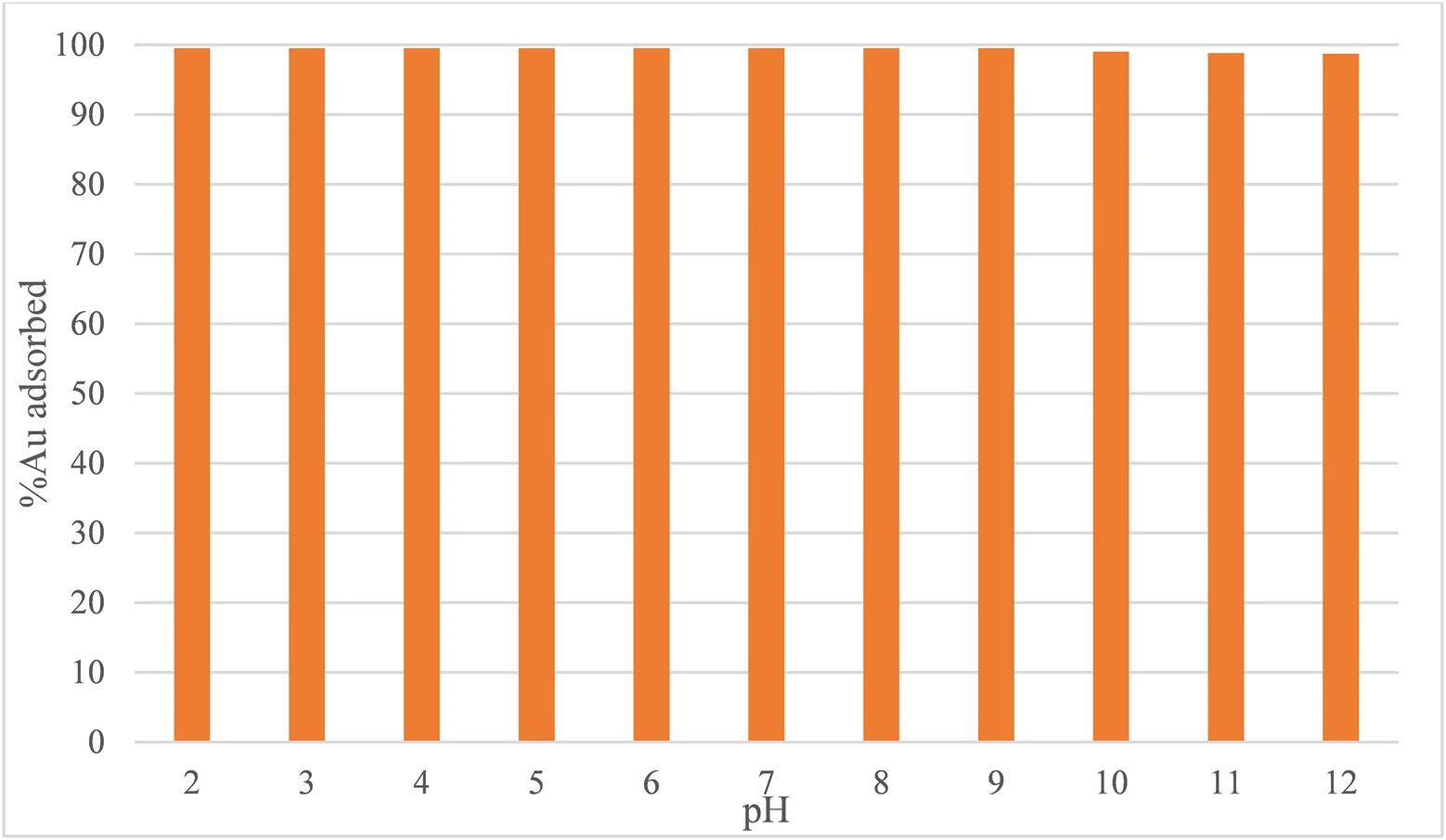
Influence of pH on the adsorption of gold onto Fe-BTC/PpPDA.

A crucial parameter that strongly influences the adsorption of metal ions is the pH. It also affects the surface properties of the adsorbent and the speciation of ions in solution. Depending on the pH of the medium, the Fe-BTC/PpPDA MOF surface exhibits different behaviours. We investigated the effect of pH on gold adsorption within a pH range of 2–12. As shown in figure 9, we observed that in the range of pH 2–10, there was no significant effect on adsorption [14]. The percentage of gold adsorbed decreased slightly at pH values above 10, indicating that Fe-BTC/PpPDA can adsorb gold in both acidic and basic media. This behaviour is attributed to the amine groups of the polymer, which coordinate gold ions beyond the point of zero charge (pHzc = 5.6). Below this potential, electrostatic interactions govern the adsorption of gold ions.

#### 3.3.4. Ligand effect

**Figure 10.**
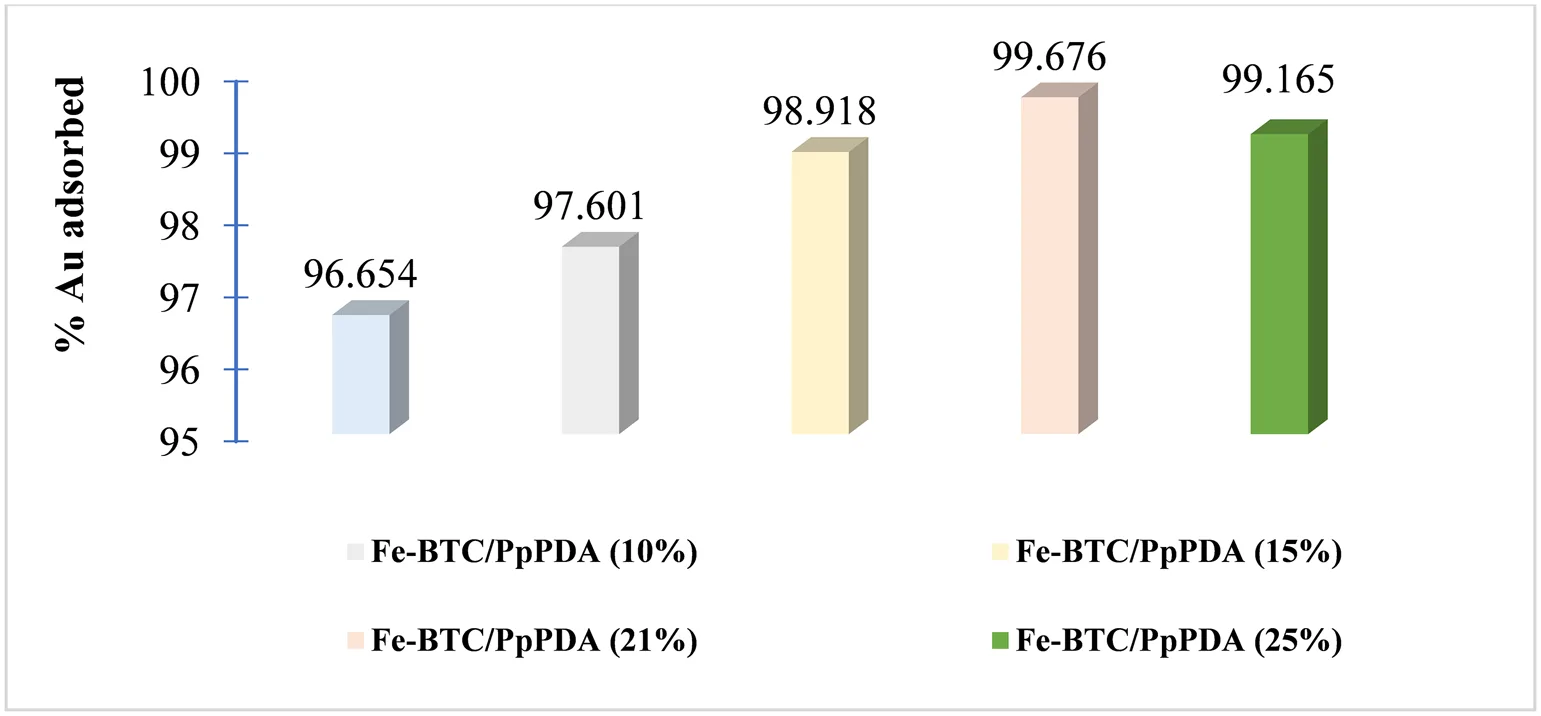
Influence of the ligand on adsorption.

A synthesis strategy involving the variation of the polymer content within the pores of Fe-BTC/PpPDA led to the formation of composite MOFs with distinct adsorption performances. As illustrated in figure 10, the adsorption capacity is influenced by the proportion of polymer embedded in the MOF structure. Notably, the composite containing 21% polymer showed the highest gold adsorption capacity [14]. This enhancement may be attributed to an optimal balance between the availability of active sites provided by the polymer and the preservation of the porous structure of Fe-BTC. At lower polymer contents, the number of functional groups may be insufficient to promote efficient interaction with gold ions, whereas at higher loadings, excessive polymer may block the MOF pores and hinder diffusion. Thus, the 21% polymer content appears to provide the best compromise for maximizing adsorption efficiency.

## 4. Conclusion

This study focuses on the synthesis and characterization of a composite material combining Fe-BTC and PpPDA. Fe-BTC was first synthesized successfully, as confirmed by various characterization techniques. The presence of Fe-O bond vibrations at 550 and 603 cm^–1^ in the IR spectrum confirmed the formation of the Fe-BTC framework. In the composite, PpPDA formation was evidenced by XRD peaks at 2*θ* = 18° and 27°, IR bands at 3422, 3156, 1569, 1035 and 803 cm^–1^ and a UV-Vis absorption band at 330 nm. These results confirm the successful incorporation of PpPDA into the Fe-BTC structure, yielding the Fe-BTC/PpPDA hybrid material. This composite combines the structural features of MOFs with the functional properties of polymers. The material was synthesized via a simple, environmentally friendly solvothermal method. Adsorption experiments revealed a high gold adsorption capacity of 1045.8 mg g^–1^, with rapid kinetics reaching equilibrium within 30 minutes. The kinetic data were best described by the pseudo-second-order model, indicating a surface phenomenon, and a diffusion coefficient of 3.05 × 10^–15^ m^2^ s^–1^ was determined, further confirming the influence of intraparticle diffusion on the overall sorption process. The equilibrium data followed the Langmuir isotherm model (R^2^ = 0.9847), indicating monolayer adsorption. Compared with conventional adsorbents, Fe-BTC/PpPDA offers superior performance in terms of capacity and environmental sustainability. These findings highlight its potential for efficient and eco-friendly gold recovery. Future work will explore regeneration performance and application to solutions containing multiple metal ions.

## Supplementary Material



## Data Availability

The datasets supporting this article, including raw adsorption data, isotherm fitting files, and characterization spectra (XRD, UV-Vis, FTIR), have been uploaded as part of the Supplementary Material [40].

## References

[1] MacGillivray LR . 2010 Metal-organic frameworks: design and application. Hoboken, NJ: John Wiley & Sons, Inc.

[2] Yang S , Li M, Guo Z, Xia N, Qu L. 2019 Facile synthesis of Fe-MOF/rGO nanocomposite as an efficient electrocatalyst for nonenzymatic H2O2 sensing. Int. J. Electrochem. Sci. 14, 7703–7716. (doi:10.20964/2019.08.42)

[3] Yap MH , Fow KL, Chen GZ. 2017 Synthesis and applications of MOF-derived porous nanostructures. Green Energy Environ. 2, 218–245. (doi:10.1016/j.gee.2017.05.003)

[4] Phuong NT , Buess-Herman C, Thom NT, Nam PT, Lam TD, Thanh DTM. 2016 Synthesis of Cu-BTC, from Cu and benzene-1,3,5-tricarboxylic acid (H3BTC), by a green electrochemical method. Green Process. Synth. 5, 537–547. (doi:10.1515/gps-2016-0096)

[5] Sanchez AC . 2014 A new synthetic method for nanoscale metal-organic frameworks and their application as contrast agents for magnetic resonance imaging. PhD thesis, Autonomous University of Barcelona, Spain.

[6] Laurier KGM , Vermoortele F, Ameloot R, De Vos DE, Hofkens J, Roeffaers MBJ. 2013 Iron(III)-based metal-organic frameworks as visible light photocatalysts. J. Am. Chem. Soc. 135, 14 488–14 491. (doi:10.1021/ja405086e) 24015906

[7] Roth J , Trukhina O, Allouss D, Stoian D, Schertenleib T, Felder T, Queen WL. 2025 Post-synthetic modification of a MOF via continuous flow methods for gold E-waste recycling. ChemSusChem 18, e202401642. (doi:10.1002/cssc.202401642) 39431488 PMC11874704

[8] Sciortino L , Alessi A, Messina F, Buscarino G, Gelardi FM. 2015 Structure of the FeBTC metal-organic framework: a model based on the local environment study. J. Phys. Chem. C 119, 7826–7830. (doi:10.1021/acs.jpcc.5b01336)

[9] Hilson G , Monhemius AJ. 2006 Alternatives to cyanide in the gold mining industry: what prospects for the future? J. Clean. Prod. 14, 1158–1167. (doi:10.1016/j.jclepro.2004.09.005)

[10] Guesh K , Caiuby CAD, Mayoral Á, Díaz-García M, Díaz I, Sanchez-Sanchez M. 2017 Sustainable preparation of MIL-100(Fe) and its photocatalytic behavior in the degradation of methyl orange in water. Cryst. Growth Des. 17, 1806–1813. (doi:10.1021/acs.cgd.6b01776)

[11] Aguiar LW , da Silva CTP, de Lima HHC, Moises MP, Rinaldi AW. 2018 Evaluation of the synthetic methods for preparing metal organic frameworks with transition metals. AIMS MaterSci. 5, 467–478. (doi:10.3934/matersci.2018.3.467)

[12] García ER , Medina RL, Lozano MM, Pérez IH, Valero MJ, Maubert Franco AM. 2014 Adsorption of azo-dye Orange II from aqueous solutions using a metal-organic framework material: iron- benzenetricarboxylate. Materials (Basel) 7, 8037–8057. (doi:10.3390/ma7128037) 28788289 PMC5456435

[13] El-Sawaf AK , Nassar AA, Elfiky AAEA, Mubarak MF. 2024 Advanced microcrystalline nanocellulose-based nanofiltration membranes for the efficient treatment of wastewater contaminated with cationic dyes. Polym. Bull. 81, 12 451–12 476. (doi:10.1007/s00289-024-05279-w)

[14] Sun DT , Gasilova N, Yang S, Oveisi E, Queen WL. 2018 Rapid, selective extraction of trace amounts of gold from complex water mixtures with a metal-organic framework (MOF)/polymer composite. J. Am. Chem. Soc. 140, 16 697–16 703. (doi:10.1021/jp212514a) 30395464

[15] Kobielska PA , Howarth AJ, Farha OK, Nayak S. 2018 Metal–organic frameworks for heavy metal removal from water. Coord. Chem. Rev. 358, 92–107. (doi:10.1016/j.ccr.2017.12.010)

[16] Ragab AH , Mettwally BS, Mubarak MF, Al-Ghamdi A, Hemdan M. 2024 Eco-friendly electrospinning of recycled nylon 6,12 waste for high-performance nonwoven nanofibers in sustainable textile applications. J. Inorg. Organomet. Polym. Mater. 34, 1491–1505. (doi:10.1007/s10904-023-02851-1)

[17] Zhu B-J , Yu X-Y, Jia Y, Peng F-M, Sun B, Zhang M-Y, Luo T, Liu J-H, Huang X-J. 2012 Iron and 1,3,5-benzenetricarboxylic metal-organic coordination polymers prepared by solvothermal method and their application in efficient as(V) removal from aqueous solutions. J. Phys. Chem. C. 116, 8601–8607. (doi:10.1021/jp212514a)

[18] Sun DT , Peng L, Reeder WS, Moosavi SM, Tiana D, Britt DK, Oveisi E. 2018 Rapid, selective heavy metal removal from water by a metal-organic framework/polydopamine composite. ACS Cent. Sci. 4, 349–356. (doi:10.1021/acscentsci.7b00605) 29632880 PMC5879484

[19] Lamelas V , Bonvalet Rolland M, Toller-Nordström L, de Oro Calderón R, Walbrühl M, Borgenstam A. 2024 Broadening of the carbon window and the appearance of core-rim carbides in WC-Fe /Ni cemented carbides. J. Alloys Compd. 999, 175078. (doi:10.1016/j.jallcom.2024.175078)

[20] Feng D , Wang K, Wei Z, Chen Y-P, Simon CM, Arvapally RK. 2018 Metal organic frameworks and ligands for MOF synthesis. Newburyport, MA: Strem Chem Inc.

[21] Elhamdi I , Souissi H, Taktak O, Kammoun S, Dhahri E, Lago E. 2024 Optical characterization and defect-induced behavior in ZnAl_1.999_ Ho_0.001_ O_4_ spinel : unraveling novel insights into structure, morphology, and spectroscopic features. Heliyon 10, e29241. (doi:10.1016/j.heliyon.2024.e29241) 38660272 PMC11040049

[22] Mubarak MF , Selim H, Hawash HB, Hemdan M. 2024 Flexible, durable, and anti‑fouling maghemite copper oxide nanocomposite‑based membrane with ultra‑high flux and efficiency for oil‑in‑water emulsions separation. Environ. Sci. Pollut. Res. 31, 2297–2313. (doi:10.1007/s11356-023-31240-x) PMC10791961 38062214

[23] Kaskel S . 2016 The chemistry of metal-organic frameworks: synthesis, characterisation and applications. Weinheim, Germany: Wiley.

[24] Elfiky AAEA , Mubarak MF, Keshawy M, El Sayed IET, Moghny TA. 2023 Removing of cationic dyes using self‑cleaning membranes‑based PVC /nano‑cellulose combined with titanium aluminate. Environ. Sci. Pollut. Res. 30, 79 091–79 105. (doi:10.1007/s11356-023-27691-x) PMC10313568 37280497

[25] Bordet A . 2016 Une nouvelle génération de nano-catalyseurs à base de carbure de fer pour le stockage chimique de l’énergie. PhD thesis, Institut National des Sciences Appliquées de Toulouse, Occitanie

[26] Remya VR , Kurian M. 2019 Synthesis and catalytic applications of metal-organic frameworks: a review on recent literature. Int. Nano Lett. 9, 17–28. (doi:10.1007/s40089-018-0255-1)

[27] Majano G , Ingold O, Yulikov M, Jeschke G, Pérez-Ramírez J. 2013 Room-temperature synthesis of Fe-BTC from layered iron hydroxides: the influence of precursor organisation. CrystEngComm 15, 9885–9892. (doi:10.1039/c3ce41366g)

[28] Flores JG , Delgado-García R, Sánchez-Sánchez M. 2022 Semiamorphous Fe-BDC: the missing link between the highly-demanded iron carboxylate MOF catalysts. Catal. Today 390–391, 237–245. (doi:10.1016/j.cattod.2021.11.004)

[29] Sanchez-Sanchez M , De Asua I, Ruano D, Diaz K. 2015 Direct synthesis, structural features, and enhanced catalytic activity of the Basolite F300-like semiamorphous Fe-BTC framework. Cryst. Growth Des. 15, 4498–4506. (doi:10.1021/acs.cgd.5b00755)

[30] Hemdan M , Ragab AH, Elyan SS, Taher MA, Mubarak MF. 2024 Eco-friendly activated carbon thin film-Zeolitic Imidazolate Framework-8 (ACTF@ZIF-8) nanocomposite for efficient methylene blue removal: synthesis, characterization, and adsorption performance. J. Clust. Sci. 36, 2. (doi:10.1007/s10876-024-02730-w)

[31] Liu Z , Chen J, Wu Y, Li Y, Zhao J, Na P. 2018 Synthesis of magnetic orderly mesoporous α-Fe2O3 nanocluster derived from MIL-100(Fe) for rapid and efficient arsenic(III,V) removal. J. Hazard. Mater. 343, 304–314. (doi:10.1016/j.jhazmat.2017.09.047) 28988056

[32] Butova VV , Soldatov MA, Guda AA, Lomachenko KA, Lamberti C. 2016 Metal-organic frameworks: structure, properties, methods of synthesis and characterization. Russ. Chem. Rev. 85, 280–307. (doi:10.1070/RCR4554)

[33] Durgaryan AA , Arakelyan RA, Durgaryan NA. 2014 Oxidative polymerization of *p*-phenylenediamine. Russ. J. Gen. Chem. 84, 1095–1100. (doi:10.1134/S1070363214060073)

[34] Adaileh AD , Ragab AH, Taher MA, Gumaah NF, Soliman MSS, Taha A, Mubarak, MF. 2025 Development of a double-shelled nanocomposite of activated carbon-nanocellulose with cationic metal oxide core for enhanced adsorption of bicarbonate from underground water. Inorg. Chem. Commun. 173, 113779. (doi:10.1016/j.inoche.2024.113779)

[35] Li T , Yuan C, Zhao Y, Chen Q, Wei M, Wang Y. 2013 Synthesis, characterization, and properties of aniline-*p*-phenylenediamine copolymers. High Perform. Polym. 25, 348–353. (doi:10.1177/0954008312465451)

[36] Du M , Li L, Li M, Si R. 2016 Adsorption mechanism on metal organic frameworks of Cu-BTC, Fe-BTC and ZIF-8 for CO_2_ capture investigated by X-ray absorption fine structure. RSC Adv. 6, 62 705–62 716. (doi:10.1039/c6ra07582g)

[37] Hussein MF , Mubarak MF, Al-Sirhani AM, Hosny R. 2024 Examining the factors that impact the formation of barite scale in water injection operations: experimental study and quantification of scale formation. Discov. Appl. Sci. 6, 519. (doi:10.1007/s42452-024-06176-7)

[38] Kari-Ferro A , Solano-Reynoso AM, Alvarez-Arias C, Echegaray-Peña NG, Choque-Quispe D. 2024 Activated polymeric materials for phosphorus removal in aqueous medium: study of kinetics and adsorption isotherm. Results Eng. 24, 103201. (doi:10.1016/j.rineng.2024.103201)

[39] Hemdan M , Ragab AH, El-Siaad HA, Kamel JK, Gumaah NF, Mubarak MF. 2024 Sustainable synthesis and environmental application of chitosan-*Ocimum basilicum* leaves-ZnO composite membrane for permanganate ion removal in wastewater treatment. Environ. Sci. Pollut. Res. 31, 66 164–66 183. (doi:10.1007/s11356-024-35612-9) 39621216

[40] Somda W , Konate FO, Koffi KR, Sanou GY, Lingane R, Bombiri IN, Khalid M, Yonli AH. 2026 Supplementary material from: Sustainable Au (III) adsorption using greenly Fe-BTC/PpPDA synthesized composite: kinetic and isothermal studies. Figshare. (doi:10.6084/m9.figshare.c.8612078)

