# Supplementary material for: Sustainable Au (III) adsorption using greenly Fe-BTC/PpPDA synthesized composite: kinetic and isothermal studies
Source: R Soc Open Sci. 2026 Sep 9;13(9):rsos251604. doi: 10.1098/rsos.251604 (PMC13561088; doi:10.1098/rsos.251604)
Supplement: Supplementary file 1 [file rsos.251604.zip › rsos.251604/rsos251604_si_001.pdf]

Supplementary material

1. Synthesis of Fe-BTC

In a Teflon autoclave (chemical reactor) containing 200 grams of water, 5 g of trimethyl-1,3,5-benzenetricarboxylate ($C_6H_3(CO_2CH_3)_3$) and 14.46 g of ferric chloride hexahydrate ($FeCl_3.6H_2O$) are introduced. The reaction mixture is loaded into a chemical reactor and heated to 150 °C for 72 hours. At the end of this period, the mixture is cooled to room temperature and vacuum-filtered using Whatman paper, rinsing thoroughly with distilled water followed by methanol. The product obtained after filtration is dried at room temperature for 24 hours and then purified by Soxhlet extraction with methanol for 24 hours. After purification, the product is dried at room temperature for 12 hours and subjected to a thermal treatment in an oven at 150 °C for 24 hours. The final result is an orange powder called Fe-1,3,5-benzenetricarboxylate (Fe-BTC).

2. Synthesis of Fe-BTC/PpPDA

In a beaker containing 3 g of active Fe-BTC (after thermal treatment), 500 mL of a methanol solution (0.02 mol/L) containing 0.802 g of para-phenylenediamine ($C_6H_8N_2$) was added. The mixture was stirred magnetically at room temperature for 24 hours. Subsequently, the mixture was vacuum-filtered using Whatman paper and thoroughly washed with distilled water followed by methanol. The solid was air-dried for 24 hours and then purified by Soxhlet extraction with methanol for 24 hours. After purification, the product was dried at room temperature for 12 hours and subjected to thermal treatment in an oven at 150 °C for 24 hours. The final product was obtained as a purple powder, referred to as Fe-1,3,5-benzenetricarboxylate/polypara-phenylenediamine (Fe-BTC/PpPDA).

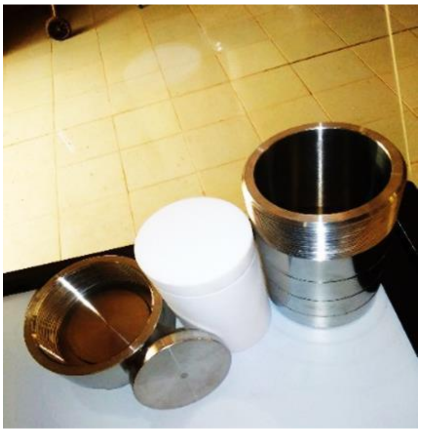

Chemical reactor using for the synthesis

3. Characterization

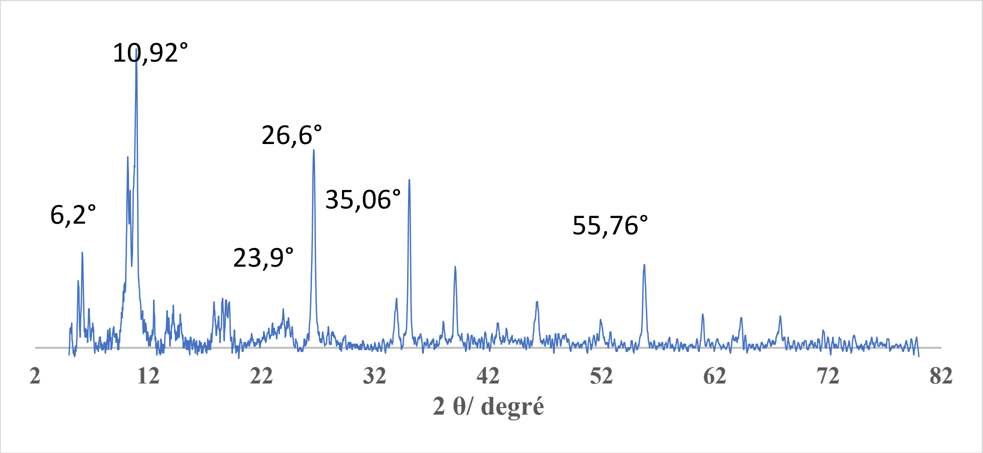

XRD pattern of Fe-BTC

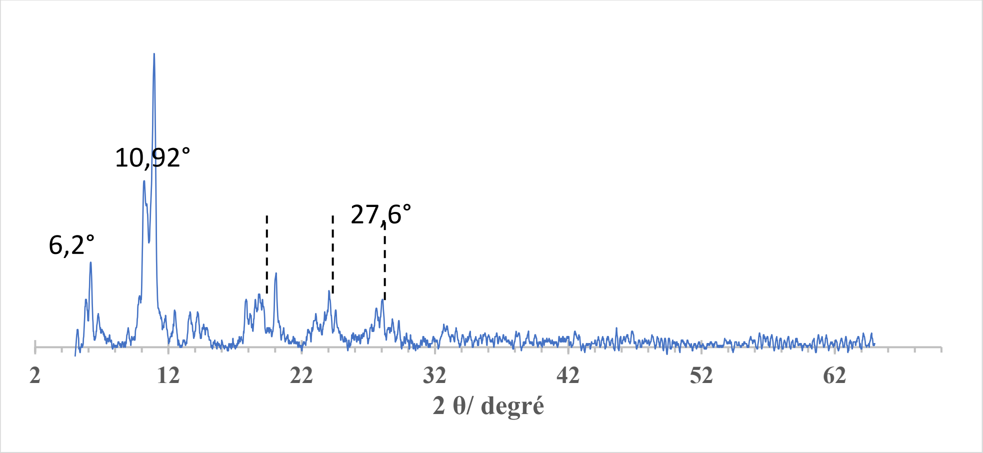

XRD pattern of Fe-BTC/PpPDA

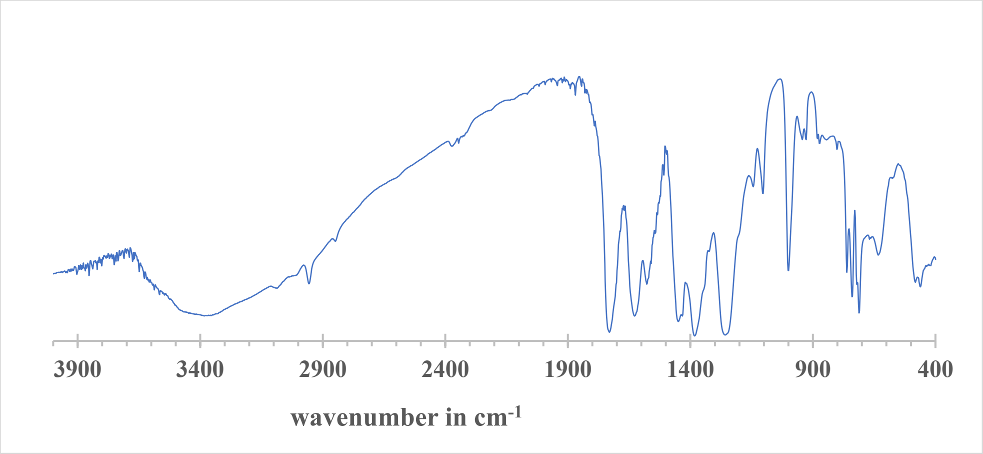

IR spectrum of Fe-BTC

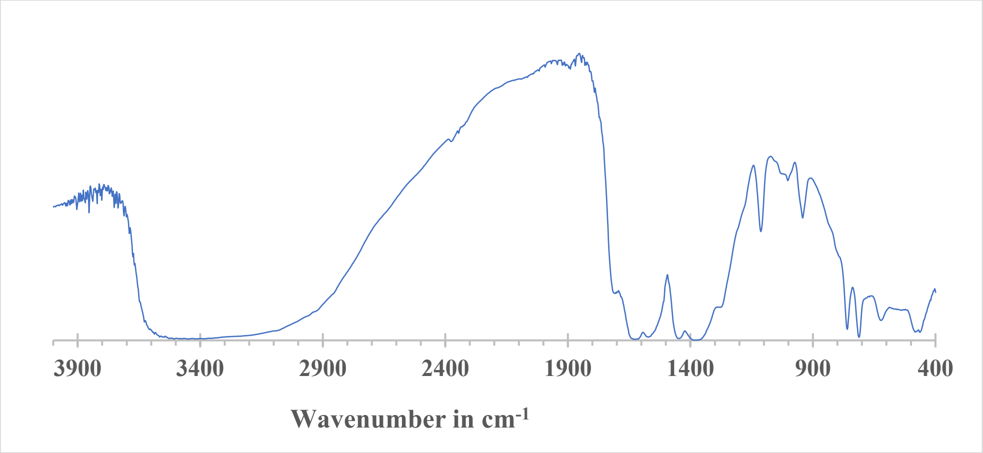

IR spectrum of Fe-BTC/PpPDA

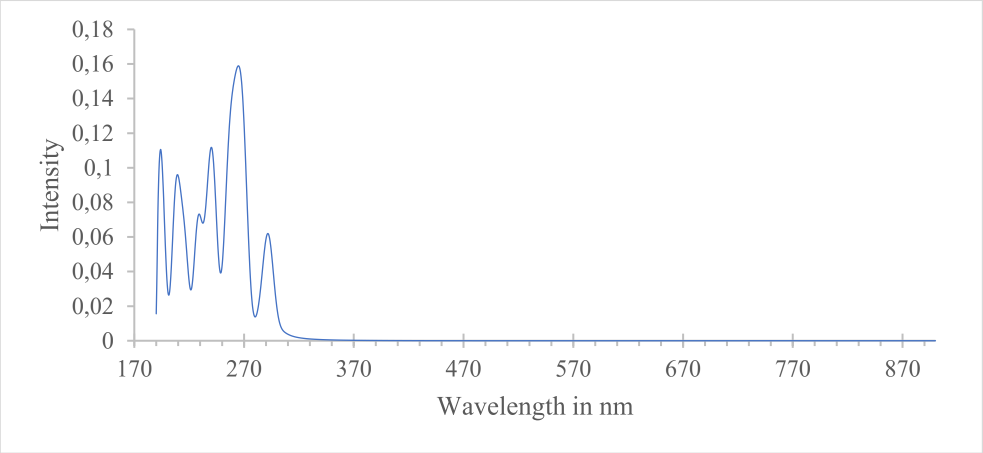

UV-vis spectrum of Fe-BTC

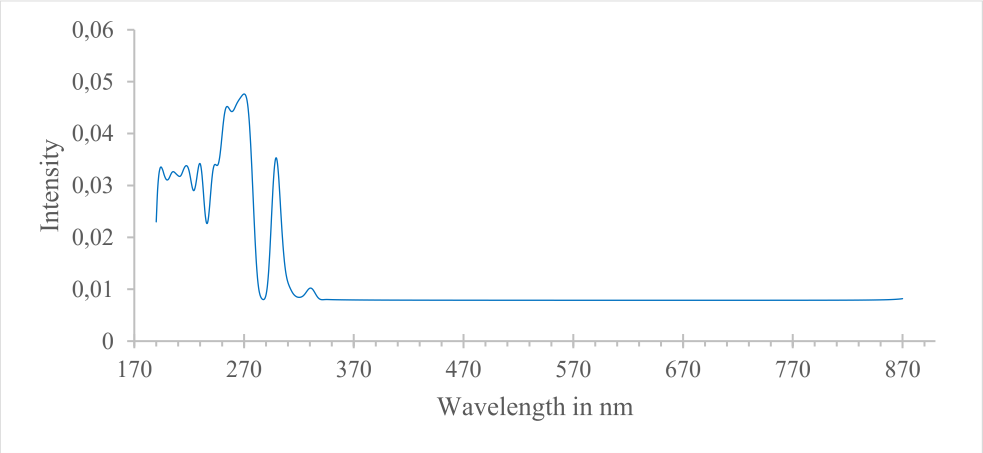

UV-vis spectrum of Fe-BTC/PpPDA

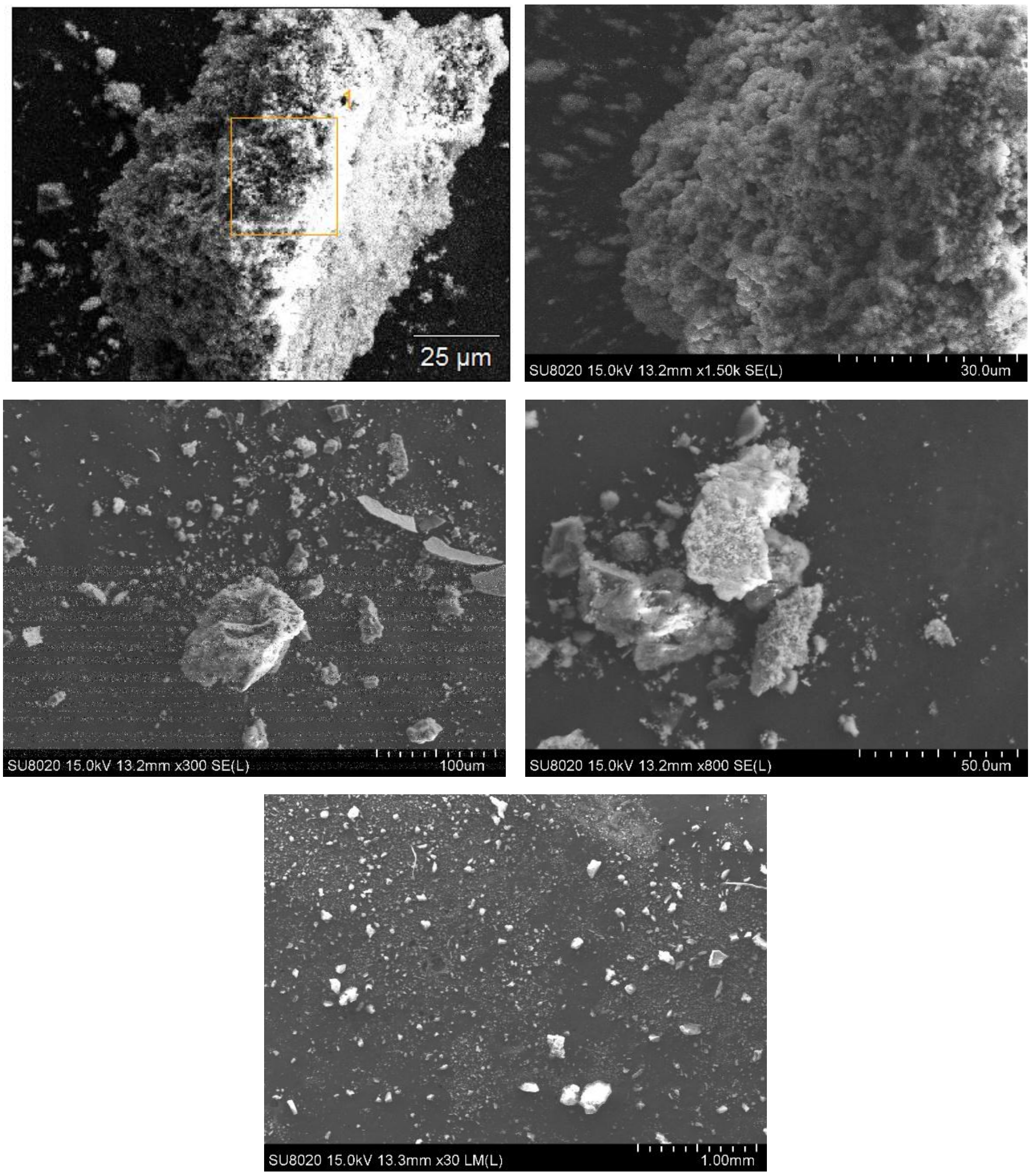

SEM images of Fe-BTC

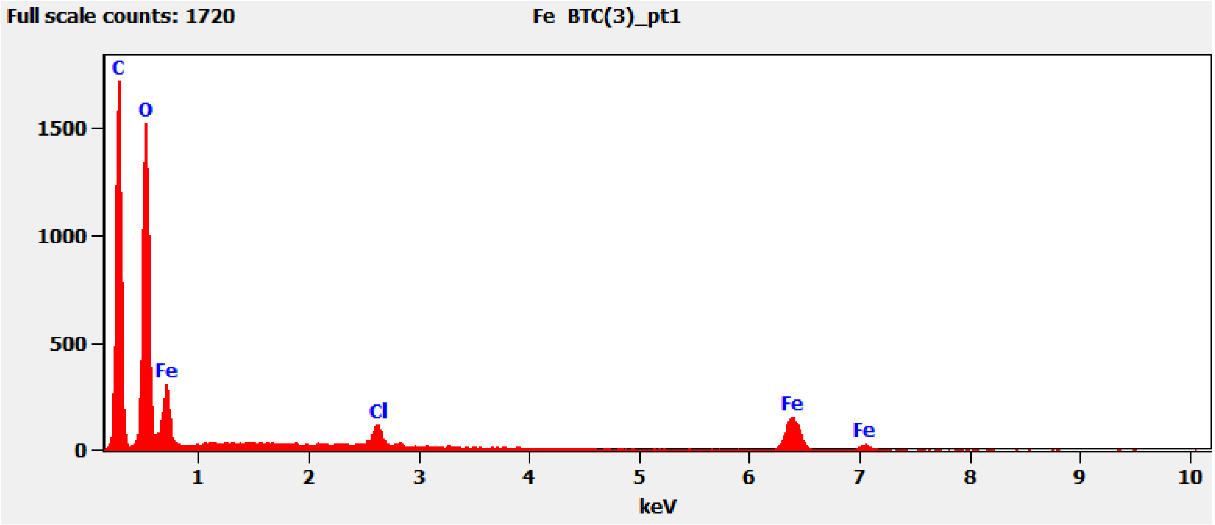

EDS of Fe-BTC

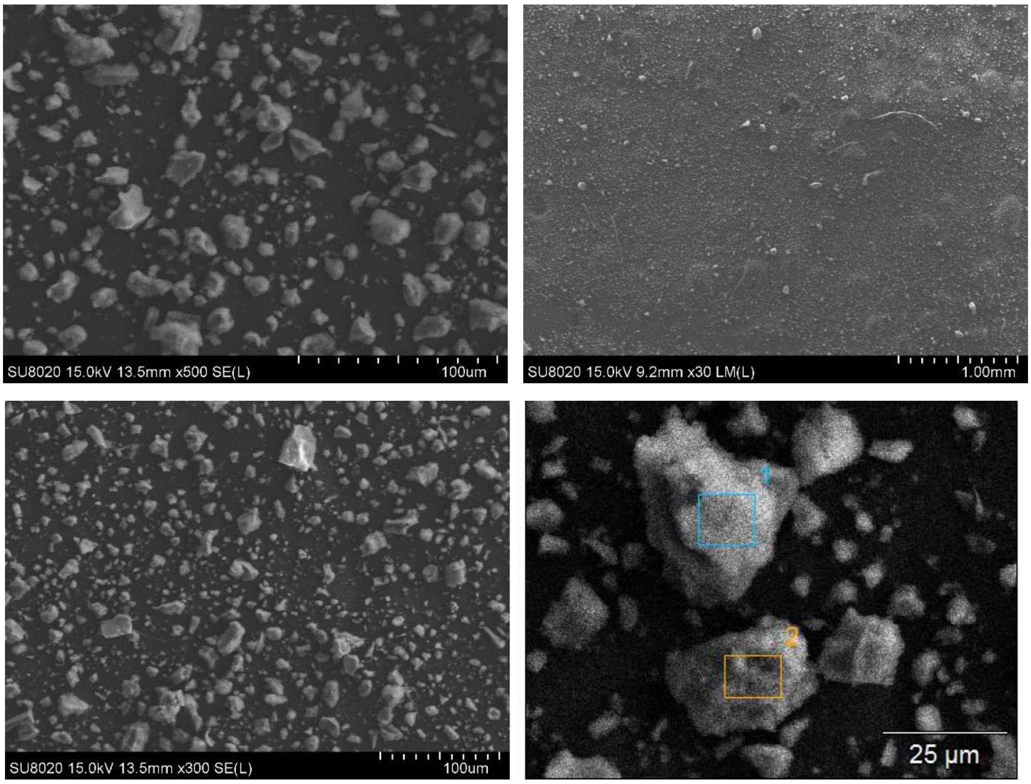

SEM image of Fe-BTC/PpPDA

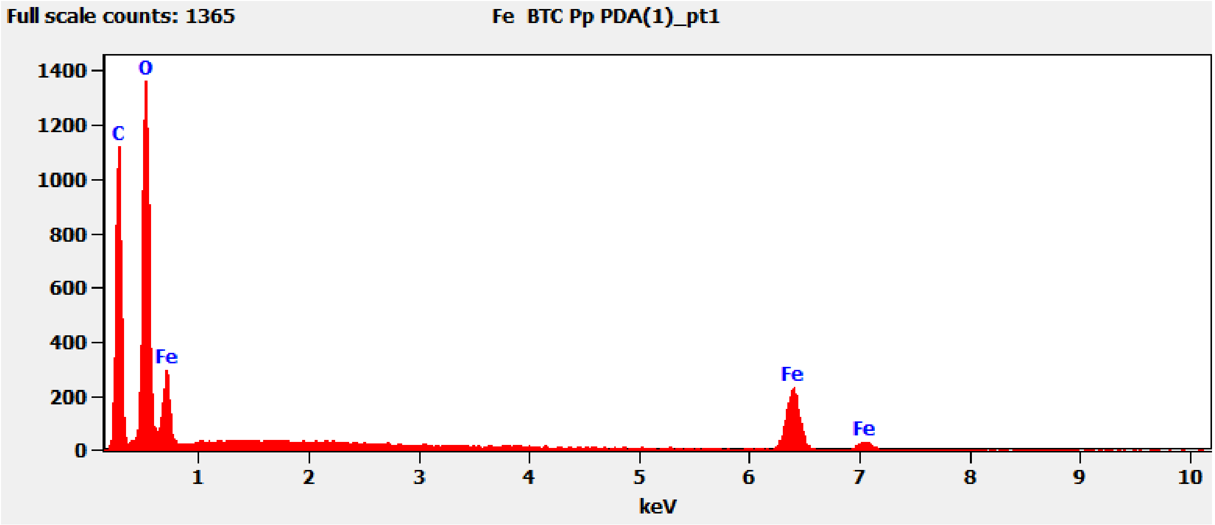

EDS of Fe-BTC/PpPDA

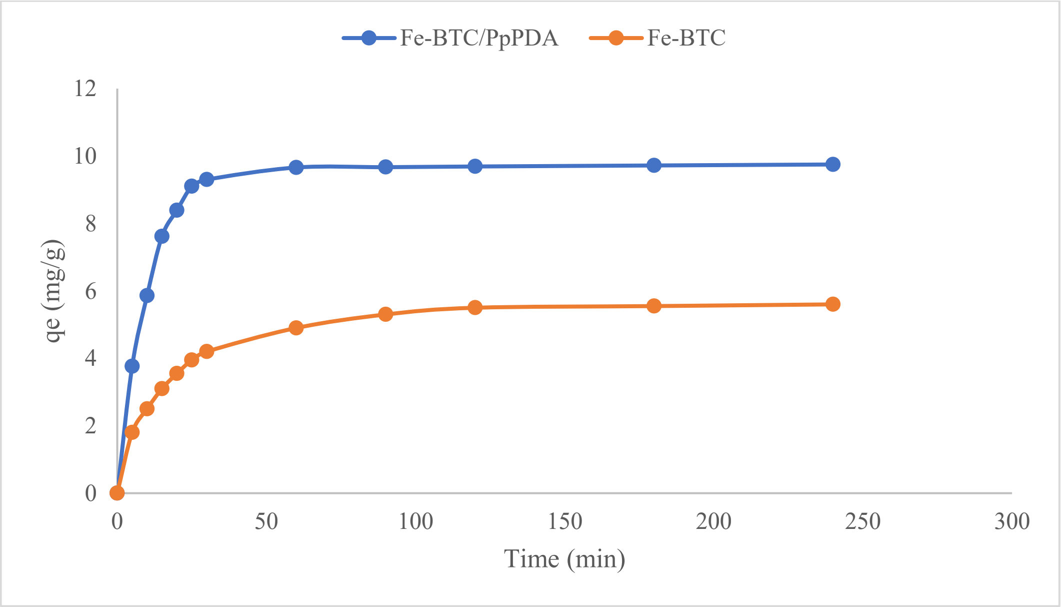

Kinetic adsorption curves of Au^{3+}

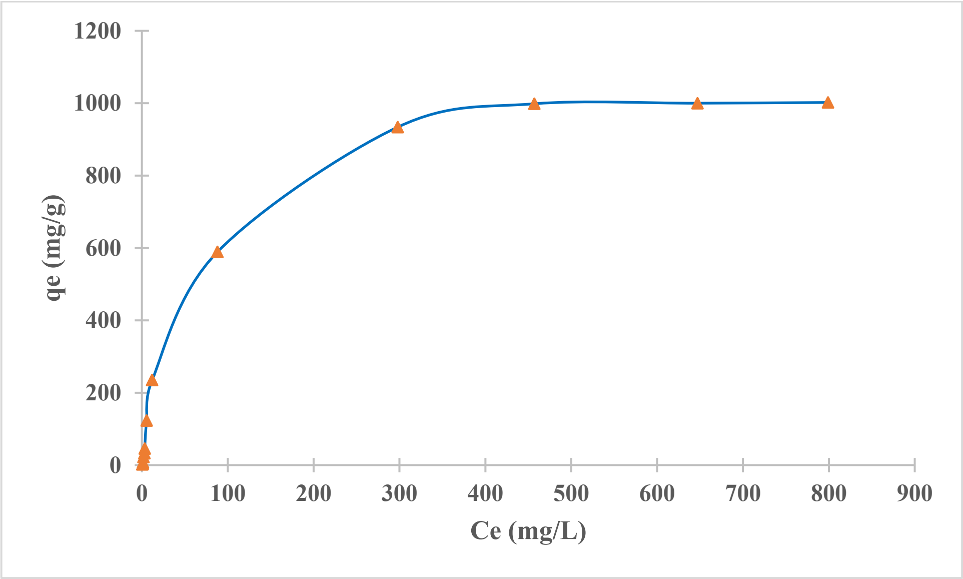

Adsorption isotherm of Fe-BTC/PpPDA

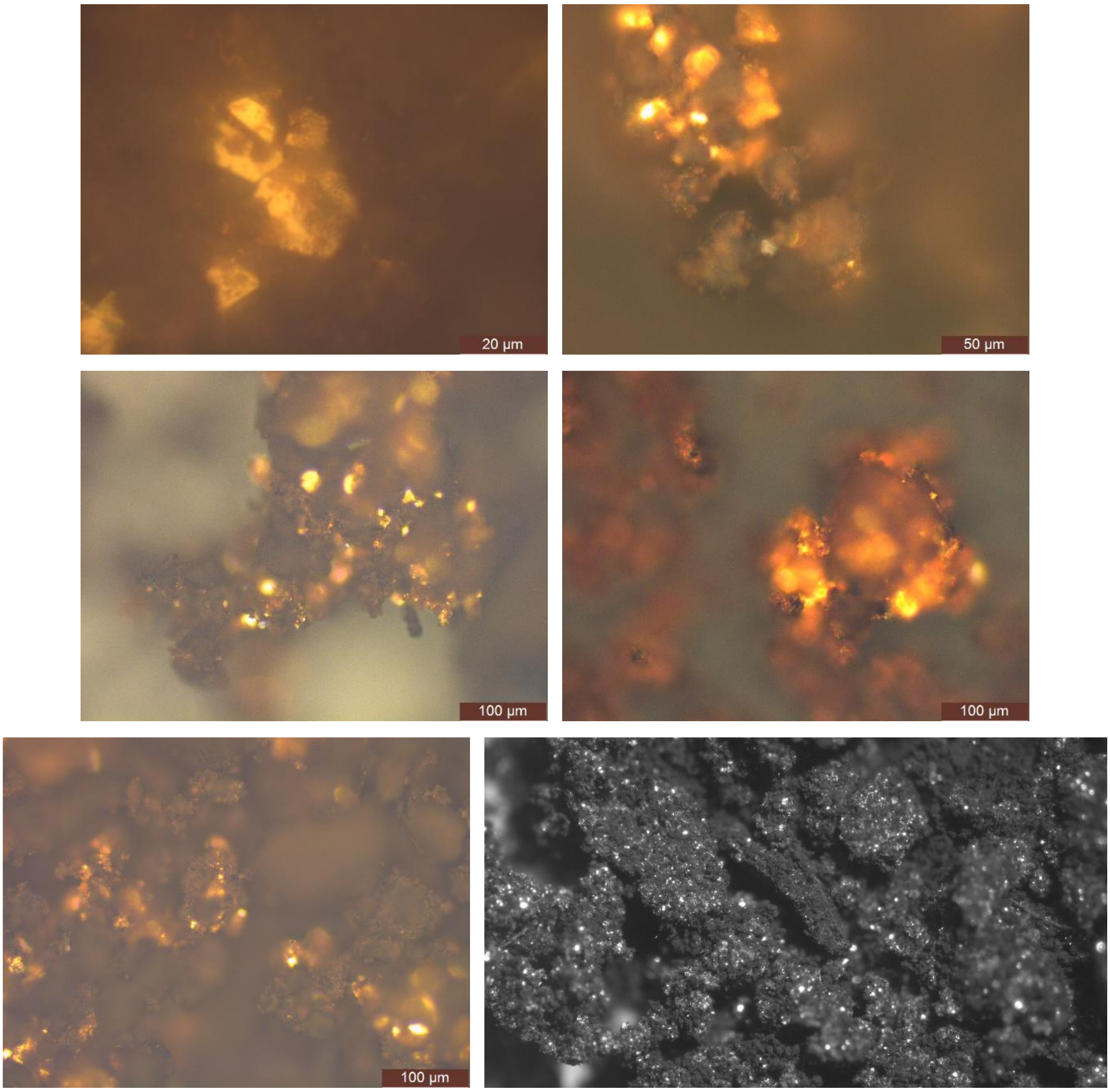

Gold adsorbed and reduced by Fe-BTC/PpPDA
